# Xanthogranulomatous Epithelial Tumor: A Case Report With 1‐Year Follow‐Up

**DOI:** 10.1002/ccr3.9675

**Published:** 2024-12-05

**Authors:** Muna AbuHejleh, Ahmed Mounir ElSayed, Renan Elsadeg Ibrahem, Asmaa Elhassan Mohamed, Adham Ammar

**Affiliations:** ^1^ Department of Laboratory Medicine and Pathology Hamad Medical Corporation Doha Qatar; ^2^ Department of Orthopedics Surgery Hamad Medical Corporation Doha Qatar; ^3^ Department of Clinical Imaging Hamad Medical Corporation Doha Qatar; ^4^ Oncology Hematology Department National Center for Cancer Care and Research (NCCCR), Hamad Medical Corporation Doha Qatar

**Keywords:** denosumab, HMGA2‐NCOR2, soft tissue and bone, xanthogranulomatous epithelial tumor

## Abstract

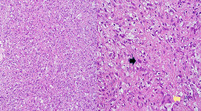

**Summary**

- Xanthogranulomatous epithelial tumor (XGET) is a rare soft tissue and bone neoplasm with distinct immunophenotypic and molecular features.
- The banal histomorphological characteristics of this lesion fail to foreshadow its potentially aggressive clinical behavior.
- The prognostic and therapeutic significance is not sufficiently explored because of the rarity of this entity.

## 1 Introduction

Xanthogranulomatous epithelial tumor (XGET) is a newly described mesenchymal neoplasm with an indolent, yet locally aggressive biological behavior, typically arising in soft tissue and bone. The diagnosis of XGET requires careful histopathological evaluation and immunohistochemical analysis to distinguish it from other xanthomatous lesions and spindle cell sarcomas. Notably, keratin expression—an unusual feature in fibrohistiocytic tumors—has been consistently observed in reported cases [1, 2, 3].

To our knowledge, only eight cases of XGET have been documented in the literature (Table 1) [1, 2, 3]. These tumors have been identified in various anatomical locations, primarily in the lower extremities and trunk, with one case exhibiting an HMGA2‐NCOR2 gene fusion [2]. Here, we present a case of XGET arising in the right acetabulum, accompanied by a 1‐year clinical and radiological follow‐up, to expand the limited understanding of this rare entity and its management.

**TABLE 1:** Cases of xanthogranulomatous epithelial tumor.

Case	Reference	Year	Age	Sex	Site	Size (cm)	Gene rearrangement
1	Fritchie et al. [1]	2020	20	Female	Calf	3.7	*PLEKHM1* mutation
2	Fritchie et al. [1]	2020	16	Female	Back	3	Not detected
3	Fritchie et al. [1]	2020	20	Female	T1–T2 Vertebrae	N/A	Not available
4	Fritchie et al. [1]	2020	22	Female	Thigh	6.5	Not available
5	Fritchie et al. [1]	2020	23	Male	Thigh	2	Not available
6	Fritchie et al. [1]	2020	62	Female	Pubic ramus	7	Not available
7	Dehner et al. [2]	2022	37	Female	Thigh	4	HMGA2‐NCOR2 fusion
8	Svantesson et al. [3]	2023	66	Female	Thigh	5	Not detected
9	Current case	2023	29	Male	Acetabulum	N/A	Not detected

## 2 Case History/Examination

**FIGURE 1:**
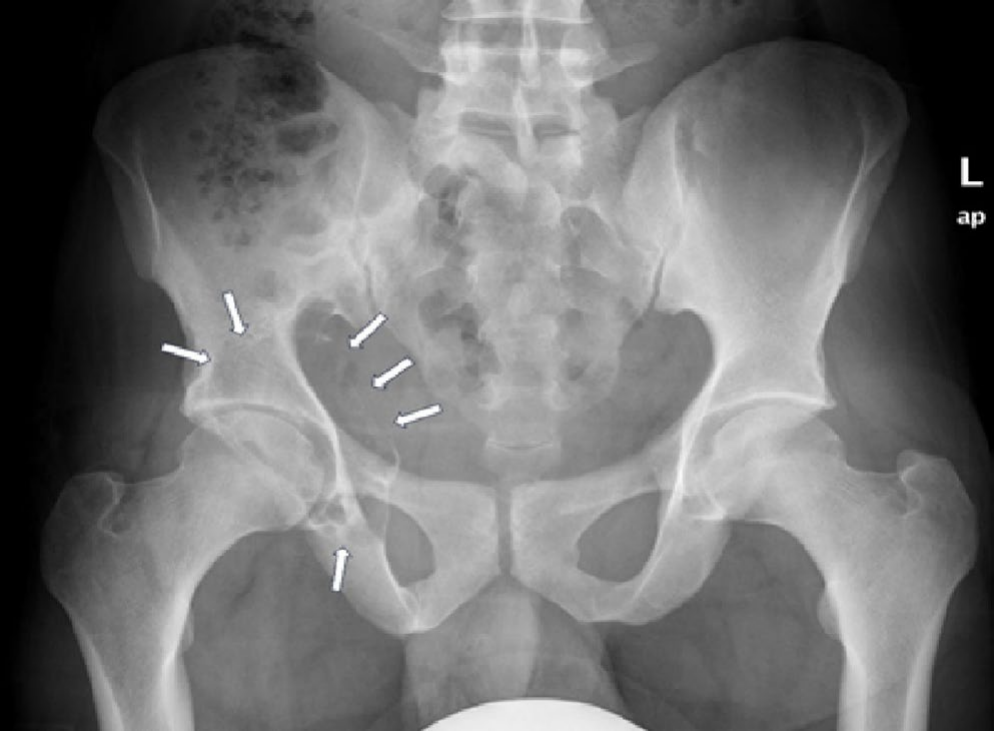
X‐ray study shows an ill‐defined expansile lesion of the right acetabulum.

**FIGURE 2:**
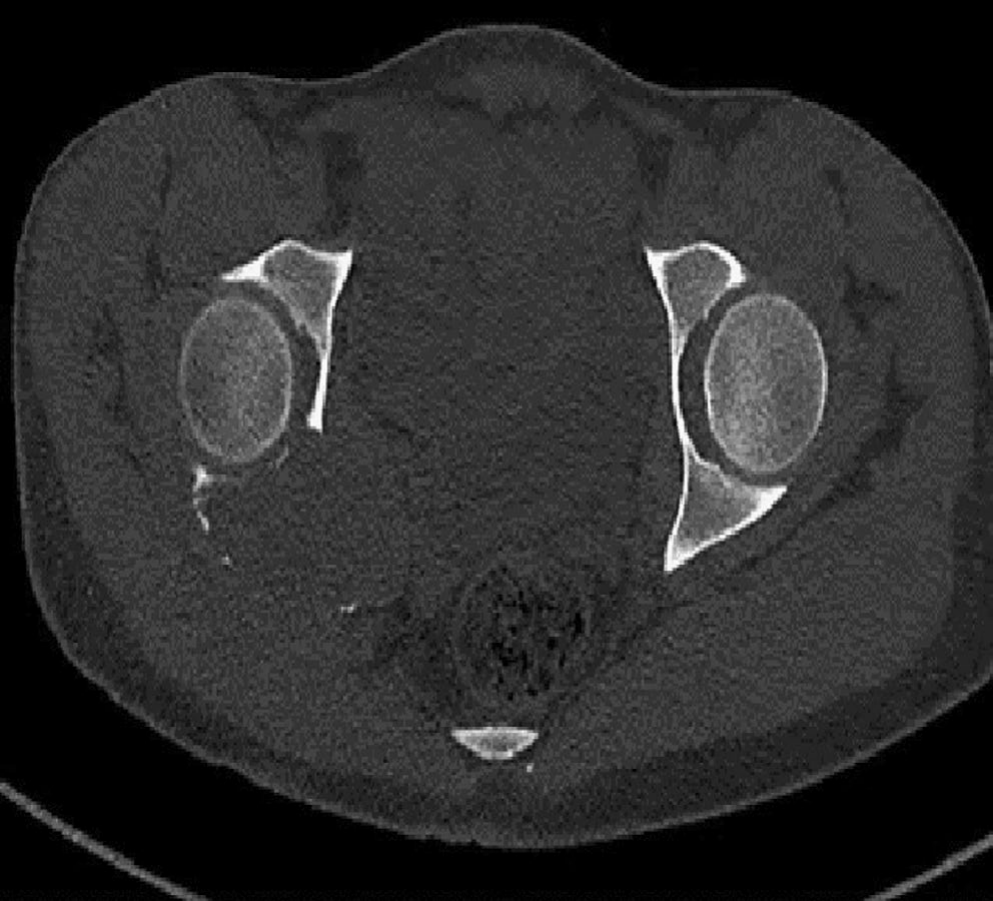
CT images of the pelvis show an expansile lytic lesion of the posterior column of the right acetabulum with breaching of the medial and lateral cortices as well as the articular surface.

**FIGURE 3:**
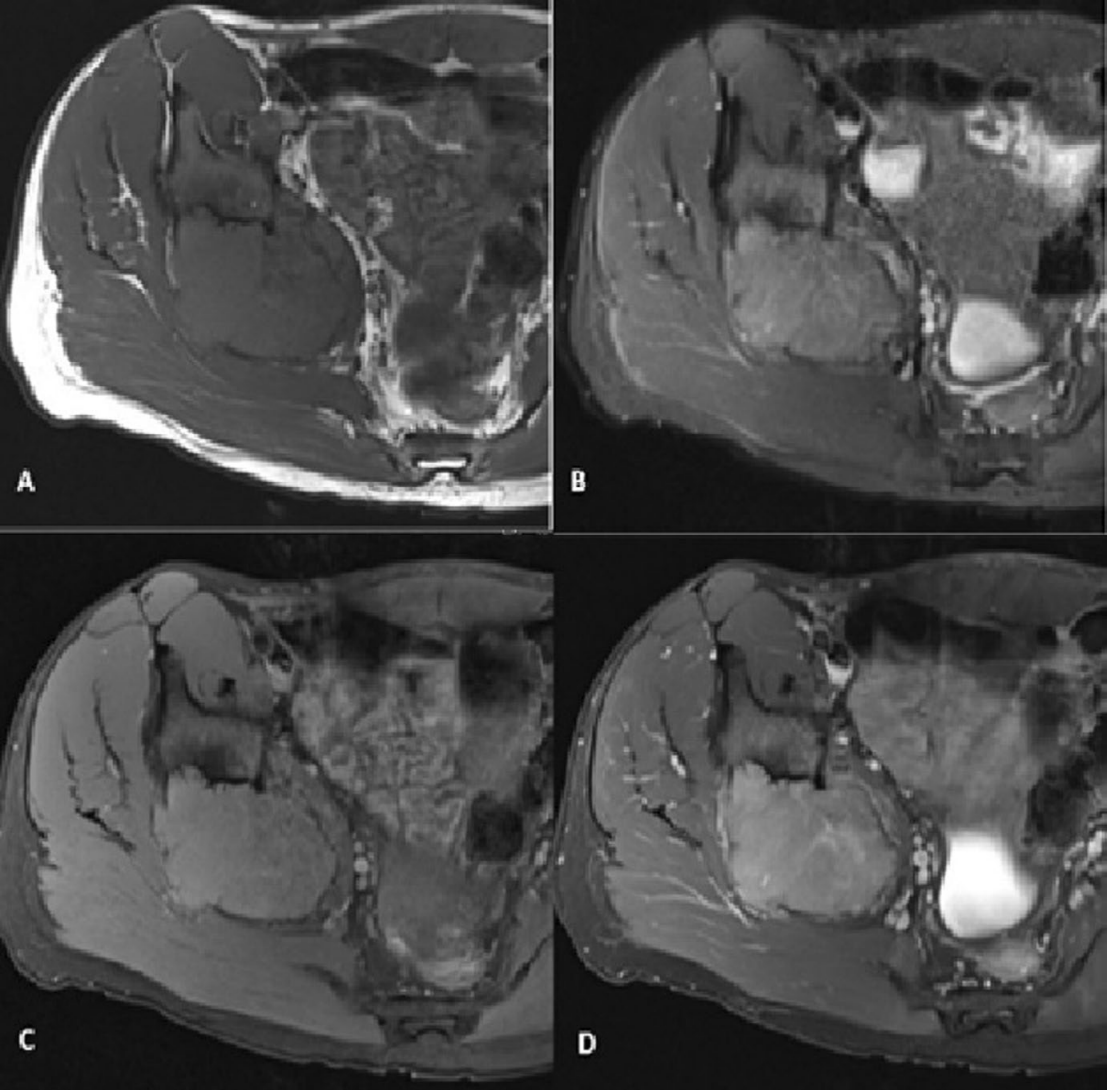
Axial MRI images of the right hip joint. (A, B) The lesion appears isointense to muscles on T1‐weighted (T1W) images and demonstrates intermediate signal intensity on STIR images. (C) Pre‐contrast T1W fat‐saturated (T1W FS) image and (D) mild diffuse enhancement on post‐contrast T1W FS images.

**FIGURE 4:**
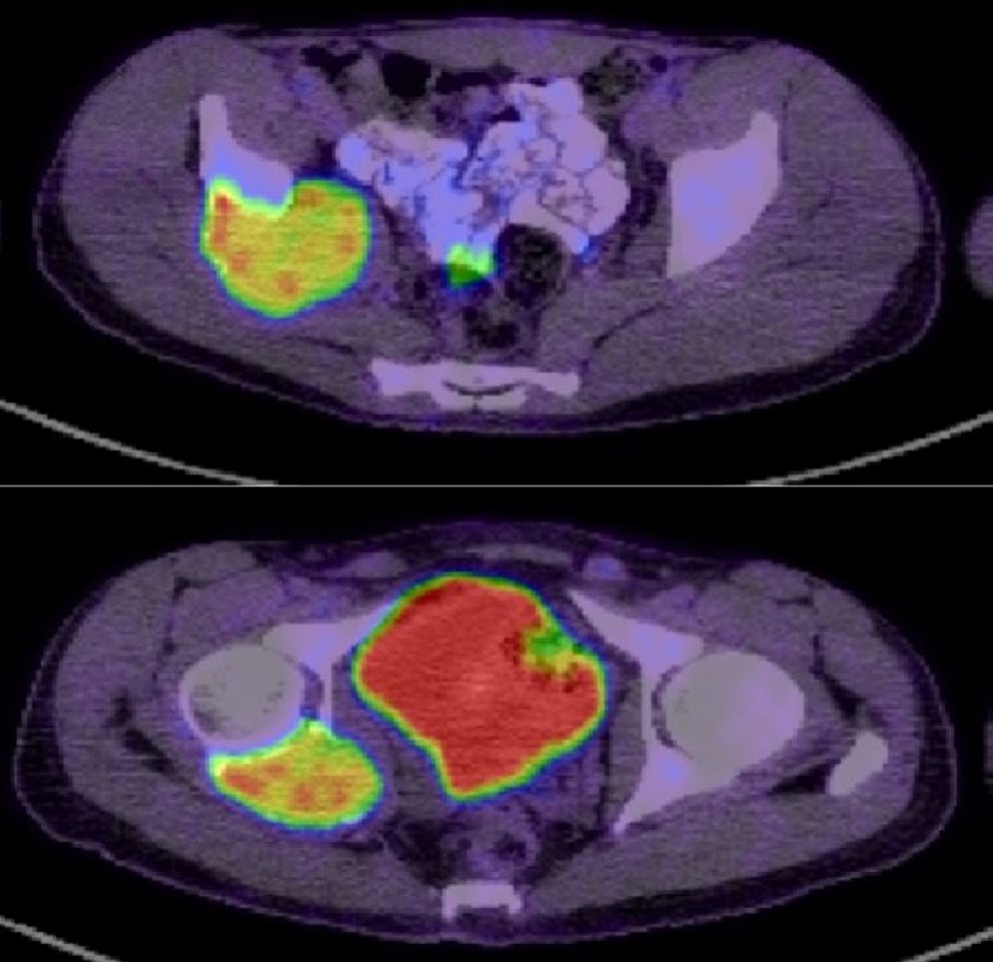
FDG PET/CT shows intensely increased tracer uptake in the expansile right acetabular/iliac osseous lesion.

A 29‐year‐old male with no previous medical or surgical history presented with right hip pain of 1 year in duration. Physical examination showed right anterior hip joint tenderness. Imaging showed an ill‐defined expansile lytic lesion of the right acetabulum centered in the posterior column and extending to involve the lower margin of the iliac bone causing complete osseous destruction (Figures 1, 2, 3). There was involvement of the acetabular articular surface. The lesion demonstrated locally aggressive radiological features and was suspicious for a malignant neoplasm. Whole‐body FDG PET/CT scan demonstrated intensely increased tracer uptake (SUV max 12) at the site of the acetabular/iliac lesion highly suggestive of malignancy. Otherwise, no abnormal uptake was seen to suggest local or distant metastasis (Figure 4).

## 3 Methods (Differential Diagnosis, Investigations, and Treatment)

**FIGURE 5:**
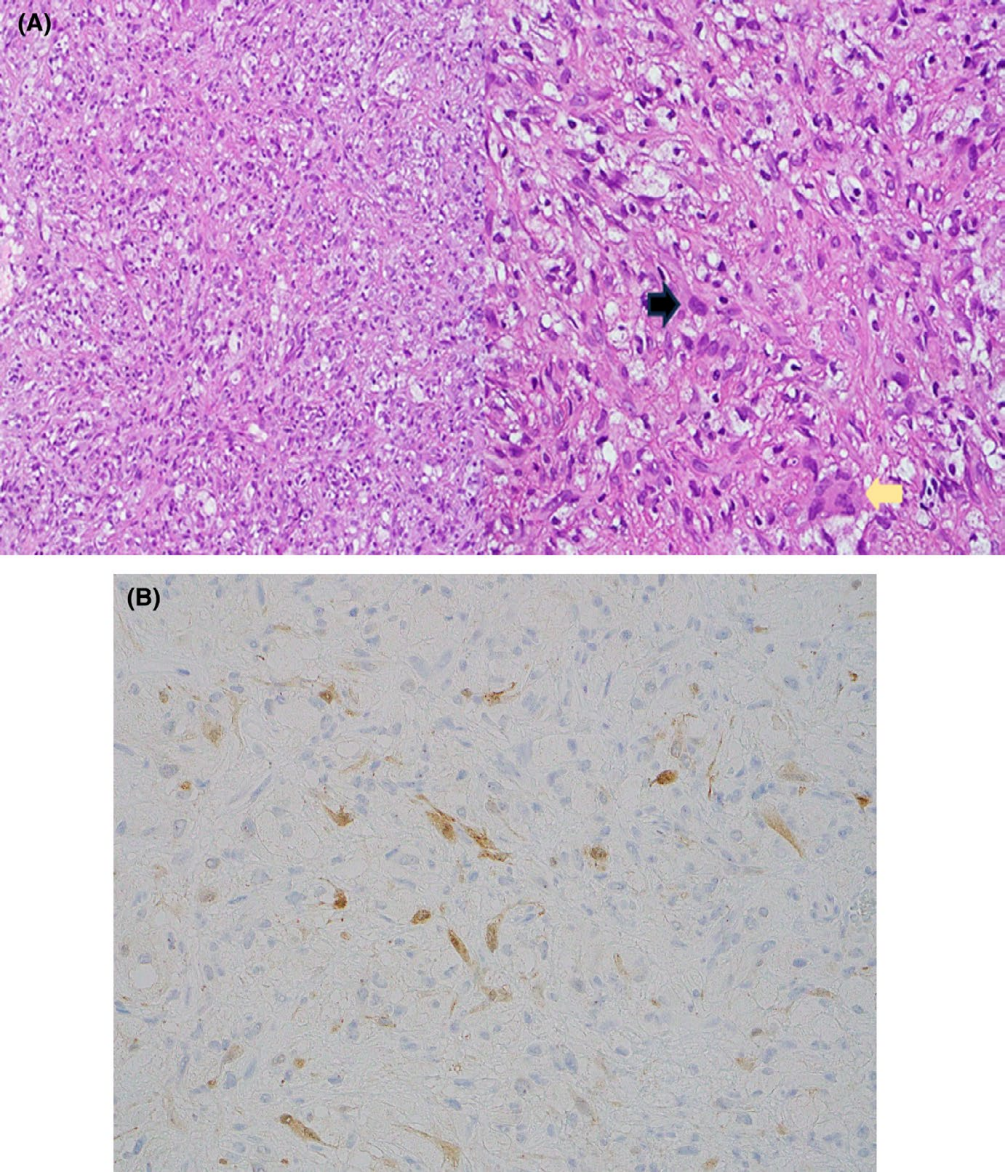
(A) Light microscopic examination reveals a tumor characterized by a proliferation of xanthomatous histiocytes with smaller, moderately atypical epithelioid cells with eosinophilic cytoplasm (black arrow). Additionally, a few osteoclast giant cells were noted (yellow arrow) (H&E stain, magnification × 100, × 400). (B) Isolated epithelioid cells were highlighted by keratin immunohistochemistry (H&E stain, × 400).

A needle core biopsy was taken from the lesion. Microscopic examination showed a prominent proliferation of xanthomatous histiocytes and smaller fibrohistiocytic cells. There were also isolated epithelioid cells with eosinophilic cytoplasm and moderate nuclear atypia. Few osteoclast giant cells were also noted. No marked pleomorphism, necrosis, or atypical mitosis was identified. No features of an overt inflammatory process nor other mesenchymal components (Figure 5A).

Immunohistochemical studies showed focal positive keratin expression in the epithelioid cells (Figure 5B). However, the cells were negative for low molecular weight keratins such as CK7, CK8/18, and CK CAM5.2. Xanthomatous cells showed diffuse positivity for CD68 and factor XIIIa. While smooth muscle, vascular, and neural differentiation markers were negative. INI‐1 (SMARCB1) immunohistochemistry retained its nuclear positivity.

The initial suggested morphological differential diagnoses were fibrohistiocytic lesion of bone, non‐ossifying fibroma, and an exuberant reaction to an adjacent neoplasm. However, focal keratin positivity and the absence of other inflammatory features ruled out those possibilities. Thus, XGET emerged as a working diagnosis. Given its rarity and scant literature, the case underwent central review at a referral center, which concurred with our diagnosis. Our case underwent a sarcoma‐targeted gene fusion panel analysis, yet no fusion was identified.

The patient initiated denosumab therapy as part of a trial to mitigate the need for extensive surgery. The patient is currently under regular follow‐up to monitor the efficacy and safety of this novel treatment approach.

## 4 Conclusion and Results (Outcome and Follow‐Up)

Our patient started denosumab therapy, by which the size of the acetabular/iliac expansile mass lesion remained stable, and after 7 months of therapy, the follow‐up PET/CT showed a significant reduction of the SUV max from 12 to 6.7. Clinically, the patient reported reduced pain with a good clinical response. Hence, he continued denosumab for another 6 months, anticipating potential surgery. One year after therapy, the patient only experienced pain upon bending, long walks, and running. MRI showed stable lesion size with slight internal changes. A recent PET/CT scan demonstrated a stable lytic lesion with mild improvement. Currently, the patient has resumed his job and reported no pain or other complaints. He continues denosumab therapy with regular follow‐ups.

## 5 Discussion

XGET is an unusual soft tissue and bone neoplasm with controversial and nonspecific radiological and histopathological features. A comprehensive literature search was conducted to identify previously reported cases of XGET [1, 2, 3]. A total of eight cases were identified, in a wide range of ages and with slight female predominance. The most common sites of involvement were soft tissue of the extremities, followed by bone. Clinical presentation varied from a painless mass to localized pain or swelling. The tumor was first described by Fritchie et al. in 2020 as an unusual mesenchymal neoplasm with indolent biological behavior [1]. Six cases were identified, arising in five females and one male with a median age of 21 years (range: 16–62). Four cases arose in soft tissue in the lower extremities and trunk. Two cases were presented in bone. In 2022, the seventh case was described by Dehner et al. a 37‐year‐old female who presented with a calf mass [2]. In a recent case report published by Svantesson et al. in 2023, they described a new case in a 66‐year‐old female who presented with a mass in her left thigh [3] (Table 1).

The pathogenesis of XGET remains uncertain, and few theories regarding the cellular origin have been proposed. In 2021, Agaimy et al. discussed the pathogenesis of keratin‐positive giant cell tumor of soft tissue (KPGCT), a rare low‐grade neoplasm that differs from conventional giant cell tumors of soft tissue (GCT‐ST) in both morphology and immunohistochemical profile. KPGCT is notable for its consistent keratin expression and should be carefully distinguished from other osteoclast‐rich, keratin‐positive tumors, such as carcinomas and epithelioid sarcomas, given its favorable prognosis. Cases reported in the literature primarily involved the head, neck, and trunk, with no recurrences or distant metastases observed [4].

The authors hypothesized that the presence of HMGA2‐NCOR2 gene fusion is potentially specific to KPGCT. To test this hypothesis, 15 cases of giant cell‐rich tumors that arose in soft tissue were analyzed. Only keratin‐positive cases harbored the distinctive HMGA2‐NCOR2 gene fusion. While keratin‐negative giant cell tumors were negative for this gene fusion [4]. Since then, Dehner et al. studied the morphological, immunohistochemical, and molecular similarities between XGET and KPGCT. Both tumors were believed to be morphological variants of a single entity. HMGA2‐NCOR2 gene fusion was detected in both neoplasms. The shared clinical, molecular, and immunohistochemical features supported the authors' theory [2].

Histological findings were consistent across all reported cases of XGET, revealing sheets of foamy histiocytes accompanied by osteoclasts and Touton‐type giant cells. Additionally, mononuclear cells with bright eosinophilic cytoplasm were observed. While necrosis was reported in one case, no marked nuclear atypia or atypical mitoses were detected [1, 2, 3].

Immunohistochemistry studies were conducted in all cases, yielding similar results. Cells exhibited at least focal positivity for keratin, CK7, and some displayed positivity for high molecular weight keratin. Additional immunohistochemistry studies, such as BRAF V600E and Histone H3G34W, were performed in some cases, all yielding negative results. Interestingly, MDM2 nuclear positivity by immunohistochemistry was observed focally in the eighth case, but no MDM2 gene amplification was detected by FISH analysis [1, 2, 3].

Molecular studies were performed in four cases. Case 1 displayed a PLEKHM1 mutation, which correlated with the patient's osteopetrosis diagnosis [1]. In the seventh case, an HMGA2‐NCOR2 gene fusion was identified [2]. However, no gene fusions were detected in the remaining cases [1, 3].

Radiological imaging studies were available for six cases (Table 2). Among soft tissue cases, the majority exhibited subcutaneous solid heterogeneous masses (Cases 1, 5, and 7) [1, 2] or well‐defined soft tissue masses with a suspected focal invasion of cortical bone (Case 8) [3]. In contrast, bone tumors demonstrated lytic lesions with sclerotic rims (Cases 3 and 6). No imaging studies were available for cases 2 and 4 [1]. No evidence of metastasis in the eight cases [1, 2, 3].

**TABLE 2:** Clinical features observed in reported cases of XGET.

Case	Radiological findings	Management	Outcome
1	Subcutaneous solid heterogeneous mass	Surgical excision	Alive with no disease
2	Not available	Excisional biopsy	Alive with no disease
3	Lytic osseous lesion with peripheral sclerotic rim	Biopsied only	Alive with disease
4	Not available	Surgical excision	Alive with no disease
5	Subcutaneous solid heterogeneous mass	Surgical excision	Alive with no disease
6	Expansile lytic lesion with peripheral sclerotic rim	Planned for excision	Alive with disease
7	Subcutaneous solid heterogeneous lobulated mass	Surgical excision	Alive with no disease
8	Well‐defined soft tissue lesion with suspected focal cortical bone invasion	Surgical excision	Alive with no disease
9^a^	Ill‐defined expansile locally aggressive lytic lesion	Denosumab trial	Good clinical and radiological response

^a^ Our current XGET case.

To date, the management of XGET poses several challenges due to its rarity and lack of established treatment guidelines. The optimal management of XGET is yet to be established. While surgical resection remains the cornerstone of therapy, the potential morbidity associated with extensive surgery underscores the importance of exploring alternative modalities.

Various management approaches were undertaken in the previously reported cases. Six of the cases were treated by surgical excision (Cases 1, 2, 4, 5, 7, and 8), one case was biopsied only (Case 3) and one case was planned for excision (Case 6). Cases that underwent complete surgical resection appear to be disease‐free upon follow‐up (follow‐up range: 3–15 months) [1, 2, 3].

Notably, a recent case study highlighted the colony‐stimulating factor 1 receptor (CSF1R) as a promising therapeutic target for XGET/KPGCT. The study found that colony stimulating factor 1 (CSF1) was overexpressed in these tumors, as demonstrated by quantitative real‐time PCR (qPCR) and chromogenic in situ hybridization (CISH). This finding suggests that CSF1R inhibitors could be a potential treatment option. However, further research and additional cases are needed to fully assess the therapeutic implications, confirm the role of CSF1R, and optimize treatment strategies for clinical application [5].

In our case, we discussed the therapeutic approach involving denosumab as an alternative to extensive surgery. Studies have explored the role of denosumab, a monoclonal antibody targeting the RANK ligand, in the management of giant cell tumors of bone [6], which share histopathological similarities with XGET. Denosumab has demonstrated promising results in reducing tumor size and alleviating symptoms in giant cell tumors, raising interest in its potential utility in other neoplasms such as XGET. However, further studies are warranted to assess the long‐term efficacy and safety of denosumab in the management of XGET.

## Author Contributions

**Muna AbuHejleh:** writing – original draft, writing – review and editing. **Ahmed Mounir ElSayed:** writing – original draft, writing – review and editing. **Renan Elsadeg Ibrahem:** writing – original draft, writing – review and editing. **Asmaa Elhassan Mohamed:** writing – original draft, writing – review and editing. **Adham Ammar:** writing – original draft, writing – review and editing.

## Ethics Statement

Written informed consent was obtained from the patient for publication of his case.

## Conflicts of Interest

The authors declare no conflicts of interest.

## Data Availability

The data that support the findings of this study are available from the corresponding author upon reasonable request.
